# Artificial intelligence-based framework for precise prediction of asphaltene particle aggregation kinetics in petroleum recovery

**DOI:** 10.1038/s41598-023-45685-0

**Published:** 2023-10-28

**Authors:** Ali Sharifzadegan, Mohammad Behnamnia, Abolfazl Dehghan Monfared

**Affiliations:** Department of Petroleum Engineering, Faculty of Petroleum, Gas and Petrochemical Engineering, Persian Gulf University, Bushehr, 75169-13817 Iran

**Keywords:** Engineering, Crude oil

## Abstract

The precipitation and deposition of asphaltene on solid surfaces present a significant challenge throughout all stages of petroleum recovery, from hydrocarbon reservoirs in porous media to wellbore and transfer pipelines. A comprehensive understanding of asphaltene aggregation phenomena is crucial for controlling deposition issues. In addition to experimental studies, accurate prediction of asphaltene aggregation kinetics, which has received less attention in previous research, is essential. This study proposes an artificial intelligence-based framework for precisely predicting asphaltene particle aggregation kinetics. Different techniques were utilized to predict the asphaltene aggregate diameter as a function of pressure, temperature, oil specific gravity, and oil asphaltene content. These methods included the adaptive neuro-fuzzy interference system (ANFIS), radial basis function (RBF) neural network optimized with the Grey Wolf Optimizer (GWO) algorithm, extreme learning machine (ELM), and multi-layer perceptron (MLP) coupled with Bayesian Regularization (BR), Levenberg–Marquardt (LM), and Scaled Conjugate Gradient (SCG) algorithms. The models were constructed using a series of published data. The results indicate the excellent correlation between predicted and experimental values using various models. However, the GWO-RBF modeling strategy demonstrated the highest accuracy among the developed models, with a determination coefficient, average absolute relative deviation percent, and root mean square error (RMSE) of 0.9993, 1.1326%, and 0.0537, respectively, for the total data.

## Introduction

Petroleum industries, either downstream or upstream, can be adversely influenced by the least understood and the most problematic portion of crude oil, known as asphaltene, as oil is being explored, produced, processed, and transported^1–4^. Asphaltenes are referred to as oil constituents that can be solved in aromatic solvents like benzene or toluene while insoluble in saturated hydrocarbons, including n-heptane or n-pentane^5,6^. As any change in the thermodynamic parameters (such as temperature, pressure, or fluid composition) occurs, small tiny-particle-based asphaltenes may develop larger and become macro particles and accordingly deposit on the solid surface^7,8^. With this in mind, precipitation and subsequent deposition of asphaltenes throughout either enhanced oil recovery techniques or natural depletion lead to severe operational and/or economic issues for oil production^9,10^. Some of the most critical consequences of asphaltene formation include adverse wettability alteration of reservoir rock, the decline in well inflow capacity, formation damage, production facilities clogging, and equipment fouling^11,12^.

Modeling and experimental research into temperature/pressure kinetics concerning asphaltene aggregation phenomena can assist in precisely predicting and/or controlling asphaltene-related issues in all stages of petroleum processing/production. In this regard, numerous laboratory studies using several experimental techniques, including the centrifugation method, small-angle X-ray and small-angle neutron scattering, confocal laser-scanning and fluorescence microscopy, optical and confocal microscopy, and particle-size analyzer were performed for analyzing asphaltene aggregation and aggregation size^13–20^. In addition to experimental studies, there have been efforts to investigate asphaltene aggregation/precipitation behavior from a theoretical perspective.

Moradi et al. studied asphaltene aggregation size distribution through miscible gas injection, which enhanced the optimum extent for collision parameter, and then employed population balance for modeling aggregation kinetic. Conclusions indicated that during natural production, cluster aggregation plays a dominant role near the bubble point pressure of crude oil. In addition, nitrogen injection significantly affects the incremental content and size of asphaltene flocs^21^.

Duran et al. investigated asphaltene aggregation size and precipitation in diluted crude oil with n-heptane using the population balance method. Results showed that collision efficiency has less impacts on precipitation aggregation^22^. Soltani Soulgani et al. showed that asphaltene collision intensity relates to particle size distribution and density of the mixture. Results indicated that increasing n-hexane concentration in asphaltene-toluene solution leads to more asphaltene aggregation. It was observed that large aggregates, due to Brownian motion, are stable in the solution for less than 200 min. In addition, the aggregation rate in the reaction-limited aggregation process was found to be quicker than that of the diffusion-limited aggregation process. Also, the average size of asphaltene aggregation decreases in the asphaltene settling region^23^. Hemmati-Sarapardeh et al. studied two main effective parameters, such as various asphaltene concentrations and normal alkane-to-toluene ratios on asphaltene aggregation. According to their findings, increasing asphaltene concentration would lead to an increase in aggregation size toward more than 100 microns, while increasing n-alkanes length leads to a decrease in the aggregation size. It was also observed that heteroatoms play a significant role in the average size of asphaltene. The asphaltene with the smallest polarity and aromaticity made a more stable solution with the lowest aggregation size^6^.

Poozesh et al. modeled asphaltene deposition in the pipeline and showed the effectiveness of medium stability and viscosity using different oil compositions. It was observed that the lower the viscosity, the greater the aggregation size and, hence, the greater the deposition rate^24^. Hosseini-Moghadam et al. used different types of chemical inhibitors of asphaltene aggregation in an undiluted dead oil. Among various types of inhibitors, dodecylbenzene sulfonic acid has the highest efficiency in reducing the aggregation size of asphaltene at all performed temperatures due to efficient acid–base interactions. Additionally, the population balance approach was utilized for modeling and analyzing aggregation size and its kinetic behavior. Their results indicated that collision efficiency is reduced with decreasing temperature and inhibitor efficiency^25^.

Despite considerable experimental and theoretical/mechanistic investigation focused on exploring the different aspects contributing to the kinetics of asphaltene aggregation behavior, some limited efforts have been made in the modeling stage of this phenomenon. In this way, the use of population balance modeling, as a widely accepted approach in the field of colloidal systems^26,27^, has been reported by some researchers^22,25^. The application of fractal theory and molecular dynamic simulation was also rarely addressed in this area^23,28,29^. In addition, the experimental measurements of asphaltene aggregation kinetics are inherently time-consuming and require interpretation procedures alongside expensive laboratory equipment. Therefore, developing a simple and efficient modeling strategy for predicting asphaltene kinetic behavior is especially important since experimental results under different conditions are scarcely found in the previously published research. To the best of our knowledge, given the importance of the availability of data for asphaltene particle aggregation, it comes as a surprise that there has been no attempt to introduce simple, efficient, and simple-to-use models to date. In this regard, the application of artificial intelligence-based approaches seems interesting.

The growing use of artificial intelligence methods is gaining significant attention, as they offer a promising means to predict established phenomena with exceptional accuracy. Artificial intelligence leverages advanced computer algorithms and machine learning models to analyze complex data patterns, excelling at managing large datasets and uncovering intricate relationships, particularly in diverse domains. In the context of predicting issues related to asphaltene deposition using various artificial intelligence methods, some instances have been discussed in the literature.

In their research, Ghorbani et al. employed a combination of a genetic algorithm (GA) and support vector regression to predict asphaltene precipitation (expressed as an amount in percentage) in terms of temperature, molecular weight, and dilution ratio^30^. Their study involved comparing the obtained results with the outcomes of two scaling equations, confirming the superior performance of their proposed approach. Hemmati-Sarapardeh and colleagues have also been involved in predicting asphaltene precipitation amount during natural depletion. They applied the Radial Basis Function (RBF) and Multilayer Perceptron (MLP) neural networks, optimized with various optimization algorithm^5^. Their analysis revealed that the most effective performance was achieved with the RBF-Particle Swarm Optimization (PSO) and MLP-Bayesian Regularization (BR) intelligent techniques. Sadi and Shahrabadi introduced a modeling scheme that integrates genetic algorithms (GA) and the group method of data handling to forecast the weight percentage of asphaltene precipitation. They assessed the accuracy of their developed approach by comparing it to the results obtained from the least squares support vector machine and scaling equations^31^.

Kardani et al. presented an RBF-ANN modeling approach aimed at estimating the weight percentage of precipitated asphaltene. Their results provided validation for the predictive capability of the developed model when compared to several previously proposed correlations^32^.

As can be inferred, most research efforts in the realm of asphaltene applications involving artificial intelligence have concentrated on predicting the amount of asphaltene precipitation. Notably, there is an apparent gap in the literature regarding investigations into the domain of aggregation kinetics.

Therefore, the main focus of the current work is to propose a new efficient, and precise framework for precise predictions of aggregation kinetics of asphaltene particles. In this regard, artificial intelligence approaches, including adaptive neuro-fuzzy interference system (ANFIS), Multi-layer Perceptron (MLP), radial basis function neural network (RBF-NN), and extreme learning machine (ELM), were employed to interrelate the average diameter of asphaltene aggregates (as output) to the input parameters: temperature, pressure, time, oil specific gravity and oil asphaltene content. The Grey Wolf Optimizer (GWO) is employed to optimize and facilitate learning in RBF-NN, while MLP modeling approach parameters are regulated using Bayesian Regularization (BR), Levenberg–Marquardt (LM), and Scaled Conjugate Gradient (SCG) algorithms. Subsequently, comparative graphical and statistical analysis is used to assess the effectiveness of the developed models in terms of goodness of fit and prediction capability.

## Model development

In essence, the effectiveness of a machine learning technique is contingent on various factors, encompassing the foundational theoretical framework and the architecture of the model (for instance, tree structures or neural networks), and the unique characteristics of the problem being addressed^33^. Additionally, the incorporation of different optimization algorithms (such as evolutionary and gradient-based) into machine learning approaches improves the tuning phase of modeling, ultimately leading to improved model performance. Moreover, the process of evaluating and comparing them plays a pivotal role in determining the most fitting modeling approach for the particular problem under consideration. Therefore, we have used these models with different theoretical bases and structures to explore the most optimized approach(s) in tackling the aggregation kinetics of asphaltene particles.

### Modeling strategies

#### Adaptive neuro-fuzzy interference system (ANFIS)

Adaptive Neuro-Fuzzy Inference System (ANFIS) developed by R. Jang^34,35^ is an intelligent algorithm based upon a hybrid neural network and fuzzy inference systems to lower the deficiencies associated with each algorithm^36,37^; in such approach, backpropagation (BP) and hybrid methods are training techniques based on data collection process that is used for training the initial FIS.

ANFIS structure is systematically similar to the fuzzy inference system developed by Takagi–Sugeno-Kang^38,39^. Gradient descent backpropagation, which is the primary learning rule in ANFIS, computes the derivative of the squared error of each output node (which is known as error rates) recursively from output to input nodes^40^. This implies a mixed learning technique combining gradient descent and least-squares computational techniques. In the forward stage, output nodes (functional signals) are processed on the way to layer 4, and consequence parameters are achieved by least squares^41^. The gradient descent then renews the premise parameters in the backward step^42^.

**Figure 1:**
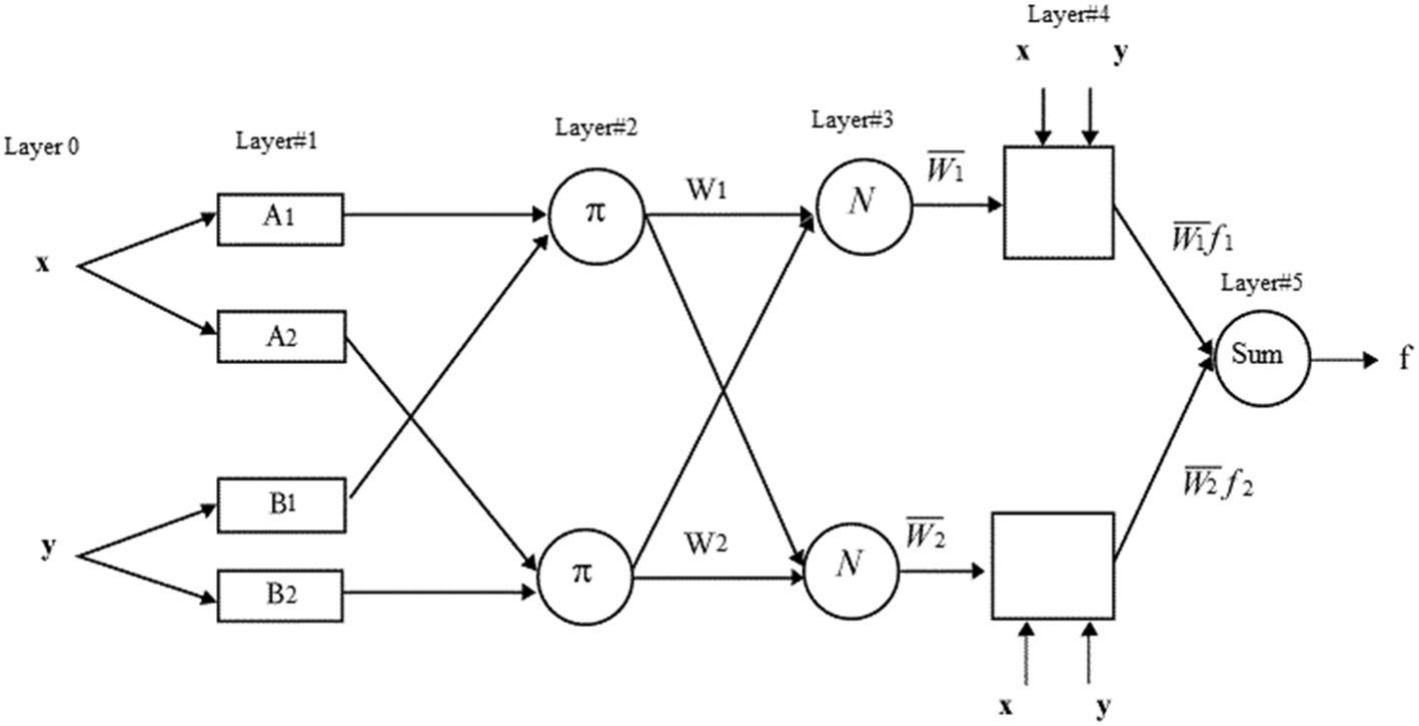
Schematic representation of ANFIS modeling approach.

The adaptive network structure is composed of 5 five network layers of 1 to 5 with nodes and connections illustrated in Fig. 1. It is believed that one output (f) and at least two inputs (which we call x and y) are considered within the fuzzy inference system (FIS).

To introduce the ANFIS architecture, two fuzzy if–then rules based upon a first-order Sugeno FIS are presented here^43,44^:

$${\text{If}}\,{\text{x}}\,{\text{is}}\,{\text{A}}_{1} \,{\text{and}}\,{\text{y}}\,{\text{is}}\,{\text{B}}_{1} \,{,}\,{\text{then}}\,{{f}}_{1} \, = \,{\text{p}}_{1} {\text{x}}\, + \,{\text{q}}_{1} {\text{y}}\, + \,{\text{r}}_{1}.$$

$${\text{If}}\,{\text{x}}\,{\text{is}}\,{\text{A}}_{2} \,{\text{and}}\,{\text{y}}\,{\text{is}}\,{\text{B}}_{2} \,{,}\,{\text{then}}\,{{f}}_{2} = {\text{ p}}_{2} {\text{x}} + {\text{ q}}_{2} {\text{y}} + {\text{r}}_{2}.$$

The two layers of this structure are defined as follows: Layer 1 defines the fuzzification process; in this layer, each node produces the membership grades of an input variable with the functions of the node explicated as follows:

1 $${\text{O}}_{{{1},{\text{i}}}} \, = \,\mu_{{{\text{Ai}}}} \left( {\text{x}} \right),\,\,\,\,\,\,{\text{i}}\, = \,{1},\,{2}.$$

2 $${\text{O}}_{{{1},{\text{i}}}} \, = \,\mu_{{{\text{Bi}} - {2}}} \left( {\text{y}} \right),\,\,\,\,\,{\text{i}}\, = \,{3},\,{4}.$$

In which O_1,i_ is the output of the node i in layer l, and subscript i denotes the membership grade of a fuzzy set that is (A_1_, B_1_, A_2_, B_2_).

The entire incoming signals are produced by the output node situated in layer 2, in accordance with:

3 $${\text{O}}_{{{2},{\text{i}}}} \, = \,{\text{w}}_{{\text{i}}} \, = \,\mu_{{{\text{Ai}}}} \left( {\text{x}} \right)\, \times \,\mu_{{{\text{Bi}}}} \left( {\text{y}} \right),\,\,\,\,\, {\text{i}}\, = \,{1},\,{2}{\text{.}}$$

The calculation for the ratio of a rule's firing strength, divided by the sum of all the rule's firing strengths, is as follows:

4 $$\normalsize {\text{O}}_{{{3},{\text{i}}}} \, = \,\overline{\text{w}}_{\text{i}}\, = \,\frac{{\text{w}}_{\text{i}}}{{{\text{w}}_1 + {\text{w}}_2}},\,\,\,\,\,{\text{i}}\, = \,1,\,2.$$

All nodes in layer 4 are the adaptive nodes with a node output:

5 $${\text{O}}_{{{4},{\text{i}}}} \, = \,\overline{\text{w}}_{\text{i}}{f}_{{\text{i}}} \, = \,\overline{{\text{w}}}_{\text{i}}\,\left( {{\text{p}}_{{\text{i}}} {\text{x }} + {\text{ q}}_{{\text{i}}} {\text{y }} + {\text{ r}}_{{\text{i}}} } \right),\,\,\,\,\,{\text{i}}\, = \,{1},\,2.$$

where q_i_, p_i_, and r_i_ are named the consequent parameters.$\overline{\text{w}}$_i_ is called normalized firing strength.

In the end, the ultimate output, which is the sum of all incoming signals, is computed in layer 5:

6 $${\mathrm{O}}_{5,\mathrm{i}} =\sum_{{\text{i}}=1}^{2}\overline{{\text{w}}}_{\text{i}}{f}_{\text{i}} = \frac{{\text{w}}_{1}{f}_{1}+ {\text{w}}_{2}{f}_{2}}{{\text{w}}_{1}+{\text{w}}_{2}}.$$

#### Radial basis function (RBF) neural network

The feed-forward network known as the radial basis function neural network, which was introduced by Broomhead and Lowe^45^, is commonly used for classification and regression tasks^33^. This method is based on the theory of function estimation, and during the training process, it transforms data into a multi-dimensional space in order to search for an optimal surface^45,46^.

**Figure 2:**
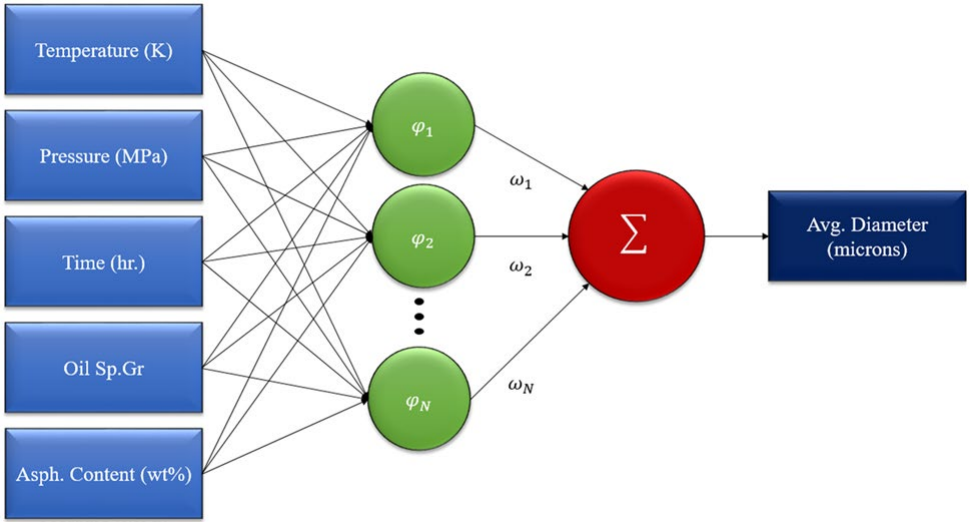
The employed RBF neural network’s specific structure.

RBF-NN comprises only three fixed layers: the input layer, the hidden layer, and the output layer^47^. The intermediate layer in RBF-NN, which is considered the most significant component, connects the input and output layers. The intermediate layer, which plays a crucial role, links the input and output layers. Each neuron in this layer is located at a specific position with an assigned radius, and the distance between the input vector and the center is then calculated^48^. The Euclidian distance is used for measuring input vectors and centers interval, which is shown in Eq. (7). Also, a simplified illustration of an RBF-NN model is shown in Fig. 2.

7 $${r}_{j}= \sqrt{\sum_{i=1}^{m}({x}_{i}-{c}_{ij}{)}^{2}} j = 1, 2,\dots , J .$$

In which ${r}_{j}$ and ${c}_{ij}$ are the radius and center.

Among all RBFs in this study, the Gaussian function was selected to transmit Euclidian distance from the hidden layer to the output. The Gaussian function is defined as follows:

8 $$\varphi \left(r\right)=\mathrm{exp}\left(-\frac{{r}^{2}}{2 {\sigma }^{2}}\right).$$

The spreading coefficient, $\sigma$, is a significant parameter that deals with the smoothness of RBF and should be quantified carefully. By using the Gaussian function as an activation function in RBF-NN, the final formulation will be obtained as follows:

9 $${y}_{i}= \sum_{j=1}^{J}{w}_{j} {\varphi }_{ij}\left(\Vert {x}_{i}-{c}_{j}\Vert \right) j = 1, 2,\dots , J \,and\, i = \mathrm{1,2}, \dots , m.$$

The RBF-NN employs weight ($w$), the number of nodes in the hidden layer ($J$), and the number of data points ($m$). The Euclidean distance is represented by $\Vert {x}_{i}-{c}_{j}\Vert$, as previously mentioned. To optimize the performance of RBF-NN, it is crucial to consider the two regularization parameters, namely, the spread coefficient of the Gaussian function and the number of nodes in the hidden layer, and optimize them simultaneously.

#### Extreme learning machine (ELM)

An extreme learning machine (ELM) is a single hidden layer feed-forward neural network (SLFN) in which input weights are selected randomly, and output weights are determined analytically in this approach. Many advantages of this method, such as high learning speed, a few adjustable parameters, proper for different non-linear problems, and generally high performance, make the algorithm more efficient^49^.

Considering *m* as the number of data points in $({x}_{i}.{y}_{i})$ form and *n* as the number of neurons in the hidden layer, this method formulation could be written as follows:

10 $${y}_{j}= \sum_{i=1}^{n}{\rho }_{i}f\left({\omega }_{i}{x}_{j}+{b}_{i}\right), j=\mathrm{1,2},3,\dots m.$$

In which ${\rho }_{i}$, $f$ , ${b}_{i}$ , and ${\omega }_{i}$ are output weights, activation function, biases, and input weights for i-th neuron, respectively. The formulation, as mentioned earlier, could be written in another way as follows:

11 $$H \rho =Y,$$

where $H$ is the hidden layer output matrix, which could be defined as follows:

12 $$H= \left[\begin{array}{ccc}f\left({\omega }_{1}{x}_{1}+{b}_{1}\right)& \cdots & f\left({\omega }_{n}{x}_{1}+{b}_{n}\right)\\ \vdots & \ddots & \vdots \\ f\left({\omega }_{1}{x}_{m}+{b}_{1}\right)& \cdots & f\left({\omega }_{n}{x}_{m}+{b}_{n}\right)\end{array}\right],$$

where, $\rho =({\rho }_{1}\dots {\rho }_{n}{)}^{T}$ and $Y=({Y}_{1}\dots {Y}_{m}{)}^{T}$^50^. The main regularization parameter in this approach is the number of neurons in the hidden layer, which is obtained empirically.

#### Multi-layer perceptron (MLP)

The most well-known classes in computational intelligence are artificial neural networks (ANNs)^51,52^. ANNs designed based on the biological nervous system of the human brain can be used in figuring out the relationships between the inputs and outputs of a system. There are two different components in each ANN, known as neurons or nods (processing element), that process information and interconnections that connect neurons. MLP and RBF are the most common ANNs. An MLP neural network has three layers: the input layer, which is related to the input data; the intermediate layer, which is called the hidden layer and plays a role between the input and output information, and finally, the output layer, which is related to the output data. In a model, the internal appearance of the relationship between the input and output is controlled by the hidden layers^53^. The number of neurons in the input layer corresponds to the number of input variables, whereas the number of neurons in the output layer typically represents the output property. It is essential to determine the number of neurons in each layer, including the number of hidden layers. Each neuron in the hidden layer is connected to all other neurons in the preceding and succeeding layers, and its value is the sum of the products of the values of each preceding neuron, a specific weight factor, and a bias term. The resulting value is then passed through activation functions, which can be applied to both hidden and output layers.

#### General characteristics of the applied AI modeling techniques

ANFIS leverages the strengths of both neural networks and fuzzy logic, making it well-suited for handling complex and non-linear systems. It exhibits flexibility in accommodating various types of data and problem domains. However, the selection of appropriate membership functions can pose a challenge, and it tends to have a slower convergence rate compared to using neural networks alone^54^. MLP exhibits remarkable proficiency in tackling intricate non-linear challenges through its utilization of multi-layer networks for modeling complex problems. Furthermore, it excels in handling substantial input data and delivering swift predictions post-training. Nevertheless, it is worth noting that MLP can be computationally demanding, and the quality of training data can significantly influence the model's function^55,56^. Like MLP, RBF can also handle nonlinear problems. The main difference between RBF and MLP lies in their structure. RBF has a simpler architecture with three layers^57^. The RBF network excels in efficient localized learning and accuracy in interpolation. However, it presents challenges in selecting the appropriate basis functions and determining the optimal number of functions. Additionally, the training phase of RBF networks tends to be computationally intensive, and they are sensitive to noisy data and high-dimensional datasets^58^. ELM offers key benefits, such as a reduced number of hyper-parameters, rapid training speed, and the ability to perform reasonably well with large datasets. Nevertheless, the main drawback of this method is limited accuracy in certain systems, as it suffers from the lack of fine-tuning for hidden layer weights^57,59^.

### Optimization algorithms

#### Levenberg–Marquardt

The Levenberg–Marquardt (LM) algorithm is an effective optimization technique that is commonly utilized in numerous applications where nonlinear least-squares problems need to be solved. Its wide usage in fields such as computer vision, machine learning, and physics is due to its capability to handle complex and noisy problems^60^. Additionally, the LM algorithm is a popular choice for optimizing biases and weights in multilayer perceptron neural networks. Notably, calculating the Hessian matrix in the LM algorithm is not required; instead, it is approximated using the equation provided below^61,62^:

13 $${H}_{m}= {J}_{m}^{T} {J}_{m}.$$

The Hessian matrix and Jacobian matrix are denoted by ${H}_{m}$ and ${J}_{m}$, respectively. In the MLP modeling approach, the Jacobian matrix is defined based on the weights and biases:

14 $$J\left(\alpha \right)= \left[\begin{array}{ccc}\frac{\partial {e}_{1}(\alpha )}{\partial {\alpha }_{1}}& \cdots & \frac{\partial {e}_{1}(\alpha )}{\partial {\alpha }_{n}}\\ \vdots & \ddots & \vdots \\ \frac{\partial {e}_{N}(\alpha )}{\partial {\alpha }_{1}}& \cdots & \frac{\partial {e}_{N}(\alpha )}{\partial {\alpha }_{n}}\end{array}\right].$$

The vector $e$ represents the errors in the network, and the equation below can be used to calculate the gradient:

15 $$g= {J}^{T} e.$$

After obtaining the gradient, the algorithm employs a Newton-like equation to update and determine the solution for the next steps, which is expressed as follows:

16 $${\omega }_{i+1}= {\omega }_{i}-({J}^{T}J- \delta I{)}^{-1} {J}^{T}e.$$

The variable $\omega$ represents the connection weight, and $\delta$ is a constant that can be adjusted during network training based on the outcome of each step. Specifically, if a step is successful, $\delta$ is decreased, whereas if a step is unsuccessful, $\delta$ is increased. Typically, the cost function decreases with each step^57,63^.

#### Bayesian regularization

The Bayesian regularization (BR) algorithm is another commonly used method in machine learning and statistical modeling that helps prevent overfitting and improve model performance. By adding a prior distribution, typically a Gaussian with zero mean and a hyper-parameter variance, to the model's parameters, the algorithm aims to identify optimal parameter values that increase the posterior probability of the model given the input data. This technique is beneficial when dealing with noisy or limited data and enables the estimation of prediction uncertainty^64^. The objective function for this algorithm is defined as:

17 $${f}_{obj}=a {\sigma }_{W}+b {\sigma }_{E}.$$

The objective function (${f}_{obj}$) is created by adding the sum of squared network weights (${\sigma }_{W}$) and the sum of network errors (${\sigma }_{E}$), with $a$ and $b$ denoting objective function parameters determined via Bayes' theorem. The BR algorithm endeavors to build a suitable network by minimizing the sum of weights and squared errors, as indicated in equation^65^. Once the optimal values for $a$ and $b$ have been determined, the algorithm employs algebraic manipulation to utilize the LM algorithm to minimize the objective function^57,63^.

#### Scaled conjugate gradient

The Scaled Conjugate Gradient (SCG) algorithm is a widely used numerical optimization method for optimizing machine learning model parameters, particularly in artificial neural networks. Its development aimed to create a more efficient and robust optimization technique than other commonly used methods, such as gradient descent and conjugate gradient. SCG combines conjugate gradient and line search techniques to minimize the objective function, and it incorporates a scaling procedure to adjust the step size based on the objective function's curvature.

The scaled conjugate gradient algorithm employs the conjugate direction for faster convergence instead of abrupt descent. The initial descent direction (${-i}_{0}$) and the search direction (${S}_{0}$), which is also referred to as the conjugate direction, are related and can be mathematically represented^61^:

18 $${S}_{0}= {-i}_{0}.$$

To identify the most suitable distance to move along the current search direction in this algorithm, a search line technique is employed. The technique can be described as follows:

19 $${u}_{j+1}= {u}_{j}+ {a}_{j} {i}_{j}.$$

The calculation of the subsequent search direction in this algorithm depends on the previous conjugate direction. The formula utilized to determine the next search direction is as follows:

20 $${S}_{j}= -{i}_{j}+ {b}_{j} {S}_{j-1}.$$

Notably, the SCG algorithm merges the conjugate gradient algorithm with the trust region approach, given that the line search method utilized to determine the step size may incur significant computational costs. Additionally, the latter is not the only approach used to determine the step size in the SCG algorithm^57,66^.

#### Grey wolf optimizer (GWO)

In this study, a meta-heuristic algorithm, namely Grey Wolf Optimizer, was utilized to optimize the RBF-NN parameters and gain more precise results. The grey wolf prefers to spend its life in the pack. The leaders are a female and a male, called alphas. The alpha is primarily responsible for decisions about hunting, sleep place, time to wake up, and so on. Beta is the second level in a gray wolf's hierarchy. The betas are obedient wolves that help the alpha in decision-making or other activities in the pack. Omega is the lowest rank of gray wolves. The omega wolf function as a submissive figure, consistently yielding to other dominant wolves. It is often referred to as a scapegoat. If a wolf does not hold the alpha, beta, or omega position, it is considered a subordinate or delta, according to some sources. Delta wolves are subordinate to the alpha and beta but dominate over the omega. The hunting behavior of grey wolves can be divided into several key stages:

- Tracking, pursuit, and approach to prey.
- Chasing, circling, and harassing the prey until it stops moving.
- Attack at the prey.

In this optimization algorithm, all of the above cases have been implemented. Additionally, the search agents have been divided into four categories: alpha, beta, delta, and omega. In this optimization algorithm, alpha represents the best solution in the current iteration. Grey wolves have a way of identifying prey's location and encircling them. Usually the alpha is in charge of hunting. Occasionally the beta and deltas would play an essential part in a hunt. Nevertheless, in the abstract search space, the best (prey) location is unknown. Mathematically modeling grey wolf hunting behavior involves assuming that the alpha, beta, and delta have superior knowledge about the potential location of prey. As a result, we save the top three best solutions gathered thus far and compel the other search agents, including the omega, to adjust their positions based on the best search agent's position^67,68^.

## Data gathering

The development of an accurate modeling strategy is strongly associated with the quality of the utilized dataset. The experimental measurements applied in the present work were collected from the literature^69^. They measured the average diameter of asphaltene aggregates for two different crude oil samples (Samples A and B) at different pressure, temperature, and time values. This dataset comprises 423 data points, of which 70% and the rest were exploited as the train, test (15%), and validation (15%) samples, respectively. The diameter of asphaltene aggregates depends on different variables such as pressure, temperature, time, characteristics of the crude oil, type of asphaltene (basic structure of asphaltene), and asphaltene content, etc. In the current study, temperature, pressure, time, oil specific gravity (as a representative of crude oil characteristics), and oil asphaltene content were considered as the input variables. The output variable is the average diameter of asphaltene aggregates. The statistical criteria of the gathered dataset are shown in Table 1.

**Table 1:** Statistical criteria for the collected dataset in this study.

Parameter	Minimum	Mean	Maximum
Temperature (K)	319	364.3026	408
Pressure (MPa)	11	32.0097	51.7
Time (hr)	0	20.8609	52.492
Oil Sp. Gr	0.85601	0.8876	0.9303
Asph. content (wt.%)	0.9	5.3681	11.4
Avg. diameter (microns)	0.4954	2.2733	17.2261

According to Table 1, each parameter has a specific range, which can decrease training process performance and, subsequently, model accuracy. Therefore, as a pre-process step the parameters mentioned above are mapped to a new range between − 1 and 1 using the equation below.

21 $${x}_{m}=2 \frac{(x-{x}_{min})}{({x}_{max}- {x}_{min})}-1.$$

## Results and discussion

### Model development

In this study, four intelligent models, namely ANFIS, RBF-NN, MLP, and ELM, are proposed to accurately calculate the average diameter of asphaltene aggregates based on various factors such as time, pressure, temperature, crude oil specific gravity, and asphaltene content. To conduct our research, we utilized a comprehensive data bank that covers a wide range of laboratory conditions.

The dataset was randomly divided into three groups, namely, the train set, the test set, and the validation set. The training set included 70% of the dataset used for training the model. The 15% of the whole dataset was selected for a test set that is utilized to evaluate the prediction capability and generality of the developed model. Also, the remaining 15% was selected as the validation set to find the optimum parameters for each model and overcome the over-fitting issues. To assess the developed models with different characteristics, some statistical parameters were calculated and subsequently compared with each other to find the most accurate one.

The optimal values of the hyper-parameters (regularization parameters) were obtained through trial and error, except for RBF-NN, where the Grey Wolf Optimization algorithm was used to find the optimal hyper-parameter values. This process was repeated multiple times to achieve the best possible results. The GWO algorithm optimized RBF-NN’s regularization parameters by 15 iterations, and the population size contains 50 search agents. The optimized values for RBF-NN, the “number of neurons” in the hidden layer and “spread coefficient,” are 99 and 0.5803, respectively. There are two popular structure types for the ANFIS modeling approach: Takagi–Sugeno-Kang (TSK) and Mamdani. In this research, the ANFIS approach was implemented using the TSK structure.

Furthermore, the FIS was generated using the Fuzzy C-Means (FCM) technique, with the parameters 'number of clusters' set to 18 and 'exponent' configured at 2.3. The exponent serves as a tuning parameter in the ANFIS algorithm, regulating the level of fuzziness, and it is conventionally assigned a value greater than 1. In the ELM modeling approach, the number of neurons has been established at 104. In the case of the MLP modeling approach, the number of neurons in the hidden layer is set to 21, and the activation functions for the hidden and output layers are Tansig. Table 2 provides a concise overview of the regularization parameter values used in the intelligent models.

**Table 2:** Optimum values for all paradigms’ regularization parameters.

Model	Regularization parameter	Value
GWO-RBF	Number of neurons	99
	Spread coefficient	0.5803
ELM	Number of neurons	104
MLP	Number of neurons	21
	Activation function	Tansig, Tansig
ANFIS	Structure of FIS	TSK
	Type of initial FIS	FCM
	Number of clusters	18
	Training method	Hybrid

### Statistical error evaluation

Statistical analysis of error parameters is an essential component of any modeling approach. To assess the reliability and performance of the developed models, several statistical indicators are commonly used. In this study, we employed three such indicators: average absolute relative deviation percentage (AARD%), determination coefficient (${\text{R}}^{2}$), and root mean square error (RMSE)^70^. These parameters are defined as follows, respectively:

22 $$\text{AARD}{\%}=\left(\frac{1}{{\text{N}}}\sum_{\text{i=1}}^{\text{N}}\left(\left|\frac{{\text{D}}^{\text{exp}}-{\text{D}}^{\text{pred}}}{{\text{D}}^{\text{exp}}}\right|\right) \right)\times100$$

23 $${\text{R}}^{2}\text{=1-}\frac{\sum_{\text{i=1}}^{\text{N}}{\left({\text{D}}^{\text{exp}}-{\text{D}}^{\text{pred}}\right)}^{2}}{\sum_{\text{i=1}}^{\text{N}}{\left({\text{D}}^{\text{exp}}-\stackrel{\mathrm{-}}{\text{D}}\right)}^{2}}$$

24 $${\text{R}}\text{MSE} = \sqrt{\frac{1}{{\text{N}}}\sum_{\text{i=1}}^{\text{N}}\left({\text{D}}^{\text{exp}}{-{\text{D}}^{\text{pred}}}\right)^{2}}$$

${\text{D}}^{\text{exp}}$ and ${\text{D}}^{\text{pred}}$ denote experientially measured and predicted values of asphaltene aggregate average diameter, respectively, and N is the number of data points.

The statistical parameters for the proposed models are presented in Table 3. The accuracy of the models was heavily influenced by the optimization of their regularization parameters. In this study, we compared the implemented paradigms based on the AARD% value, as it is not affected by the scale of the data.

**Table 3:** Different statistical parameters for proposed paradigms.

Model	Statistical parameter	Training data	Validation data	Testing data	Total data
ANFIS	AARD%	1.4998	1.5395	1.9146	1.5674
	R^2^	0.9993	0.9985	0.9982	0.9992
	RMSE	0.0578	0.0536	0.0766	0.0604
GWO-RBF	AARD%	1.0742	1.265	1.2759	1.1326
	R^2^	0.9996	0.9988	0.9990	0.9993
	RMSE	0.0409	0.0834	0.0675	0.0537
MLP-LM	AARD%	1.1015	1.3800	1.3312	1.1772
	R^2^	0.9997	0.9981	0.9974	0.9993
	RMSE	0.0415	0.0519	0.0967	0.0548
MLP-BR	AARD%	1.1535	1.5675	1.5953	1.281
	R^2^	0.9997	0.9978	0.9985	0.9992
	RMSE	0.0349	0.0961	0.0853	0.0575
MLP-SCG	AARD%	2.6824	3.0387	3.1918	2.8113
	R^2^	0.9977	0.9947	0.9977	0.9972
	RMSE	0.0941	0.1559	0.1219	0.1098
ELM	AARD%	1.7356	–	2.7408	2.0374
	R^2^	0.9996	–	0.9946	0.9980
	RMSE	0.0385	–	0.1611	0.0940

### Graphical error evaluation

**Figure 3:**
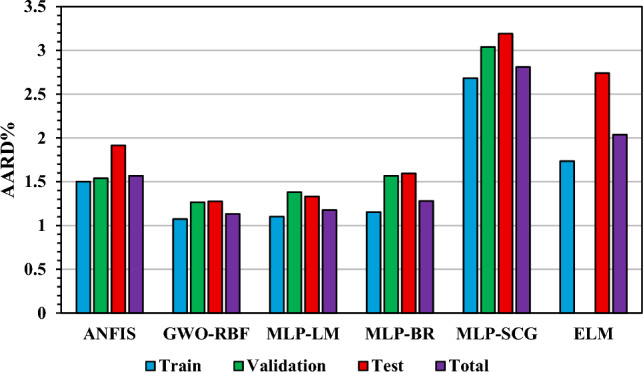
AARD% value for train, validation, test, and total set.

Statistical plots such as Cumulative frequency versus absolute relative error plot, cross plot, average absolute relative deviation percent plot, Relative error versus experimental data points plot, and error distribution plot are practical tools to evaluate the performance of the developed models visually. Figure 3 illustrates the average absolute relative deviation percent (${\text{AARD}{\%}}$) for train, validation, test, and total sets.

**Figure 4:**
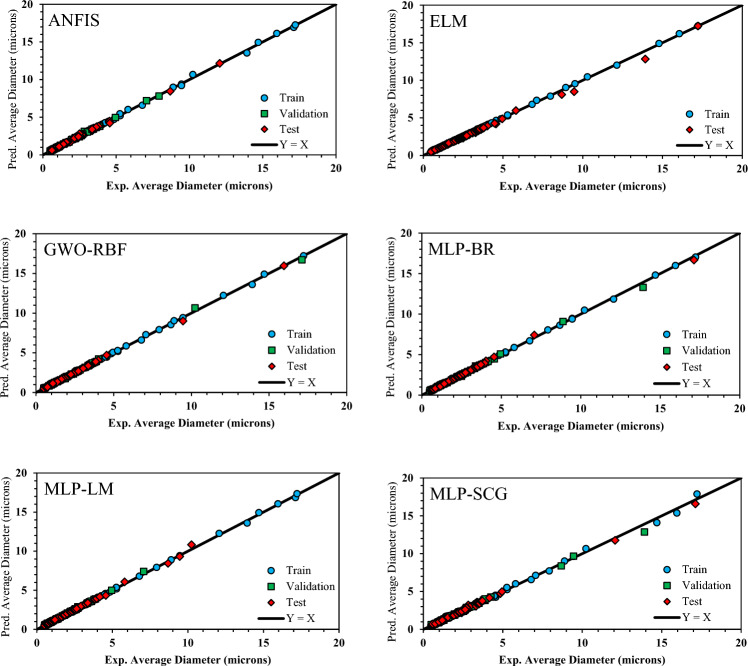
Predicted versus experimental values of average asphaltene aggregate diameter.

Based on the AARD% values presented in Table 3 and Fig. 3, all models in this study exhibit satisfactory performance. However, it is evident that the GWO-RBF model provides the best results in terms of statistical parameters, while the MLP-SCG approach demonstrates the lowest prediction capability. Figure 4 displays cross-plots of predicted and experimental data points for the train, validation, and test sets. The one-slope line that emerged from the data points for each model confirms their accuracy.

**Figure 5:**
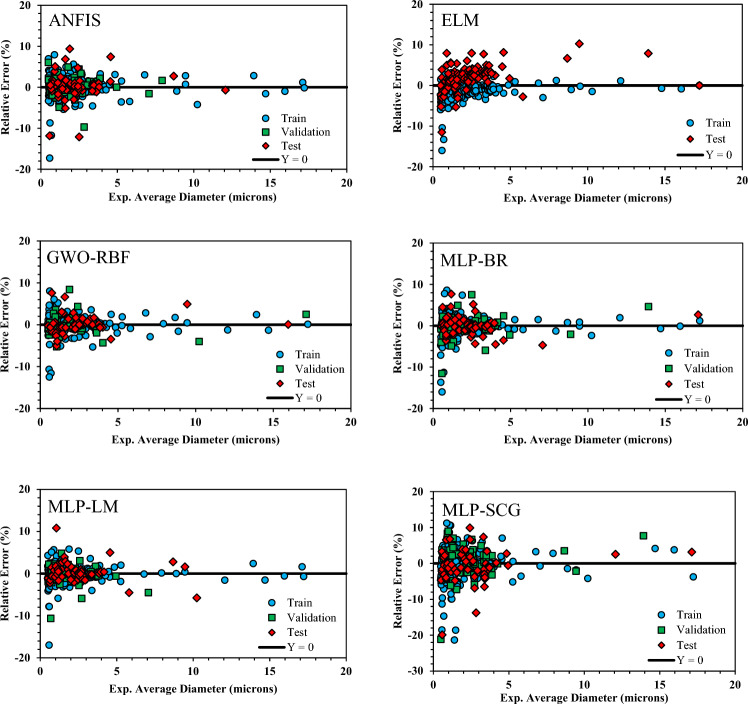
Relative error versus experimental average diameter.

Figure 5 illustrates the relative error percent (RE %) versus the experimental average diameter. It is worth noting that the accumulation of data points around the zero error line indicates the model’s superiority. Figure 5 illustrates that all proposed approaches exhibit a satisfactory clustering of data points around the zero error line. However, certain data points with low average diameter may not provide as accurate predictions as others. Among the models tested, GWO-RBF displays the most pronounced concentration around the zero error line.

**Figure 6:**
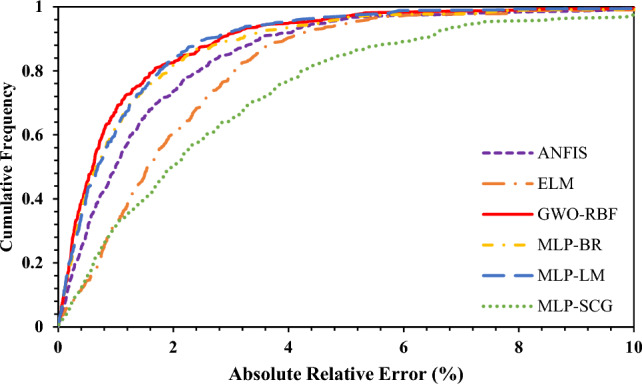
Cumulative frequency versus absolute relative error.

Additionally, Fig. 6 depicts the cumulative frequency versus absolute relative error for all paradigms investigated in this study. As shown in this figure, for ELM, ANFIS, MLP-BR, and MLP-SCG models, 90.31%, 91.73%, 93.38% and 76.83% of the whole data have an absolute relative error of less than 4%, respectively. In the same condition, this figure indicates more than 94% for the GWO-RBF and MLP-LM paradigms. Also, it should be noted that the maximum value of absolute relative errors for GWO-RBF, ANFIS, ELM, MLP-BR, MLP-LM, and MLP-SCG are 12.50%, 17.31%, 16.03%, 16.02%, 16.99%, and 21.36%, respectively. Therefore, based on previous explanations, GWO-RBF can be selected as the best model in this study, and all of the paradigms can be ranked as follows:

$$GWO-RBF>MLP-LM>MLP-BR>ANFIS>ELM>MLP-SCG$$

**Figure 7:**
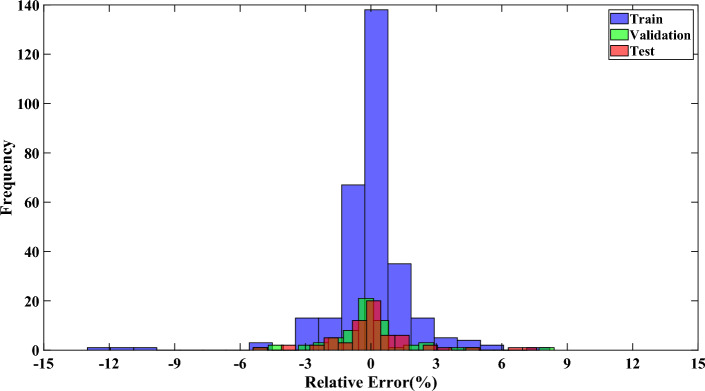
Comparison of data frequency versus relative error percent for all data points for GWO-RBF.

Figure 7 further confirms the robustness of the best-developed model. This figure illustrates the relative error percent (RE%) between the predicted and experimental values of the GWO-RBF model. As shown in the plot, the majority of predicted results by the best-developed model have relative error values within the range of − 3% to + 3% for the entire dataset. This indicates the high accuracy of the presented model.

In addition to accuracy comparison, the machine learning approaches employed can also be compared based on their computational effort. In this regard, the models were evaluated in terms of their CPU time consumption. It is important to mention that the model codes were executed on a computer system equipped with a Core i7-4910MQ processor, running at a base frequency of 2.90 GHz.

**Figure 8:**
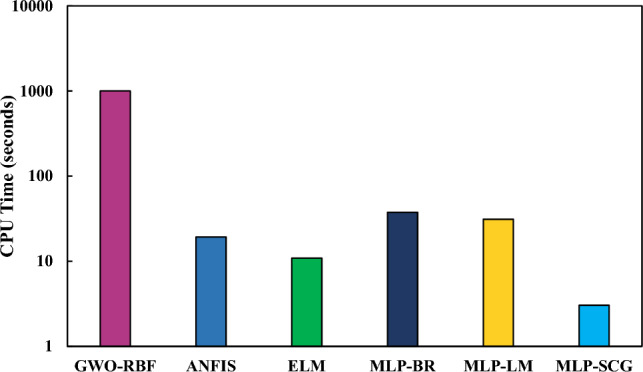
CPU time for applied machine learning schemes.

Figure 8 provides a comparison of different methods based on CPU time. As shown, the GWO-RBF (which performed the best based on the AARD%) exhibited the highest CPU time among the applied techniques. The reason for the increased CPU time for the GWO-RBF method, compared to other methods, is its utilization of a metaheuristic optimization algorithm to find optimal solutions for two hyper-parameters within this model. This metaheuristic algorithm requires more time to converge to optimal values as it operates on a population-based approach. However, it is worth noting that such algorithms tend to achieve high-quality solutions.

In contrast, MLP-SCG showed the shortest CPU time. This can be attributed to the SCG algorithm, which operates based on gradients, enabling it to converge more rapidly than the GWO algorithm, which relies on a population-based meta-heuristic approach. However, it is worth noting that gradient-based algorithms are more susceptible to becoming trapped in local optima when compared to meta-heuristic methods.

Overall, in scenarios where computational resources are limited, lightweight and efficient models (e.g., MLP and ELM) can be applied with an acceptable degree of prediction error. Conversely, when computational resources are more abundant, and a strong emphasis is placed on achieving high accuracy, it becomes viable to utilize models that offer higher accuracy at the expense of increased resource consumption, such as GWO-RBF.

### Computational complexity of models

Assessing the computational complexity of different machine learning models can be a laborious task. This is because it hinges on several factors, including the way the model is structured, the size of the dataset, the optimization methods employed, and so on. In this way, big O notation is used to explain the worst-case behavior for an algorithm complexity. Furthermore, the big O notation typically considers all feature inputs collectively, while the number of samples may fluctuate throughout the analysis^71^. Although the exact determination of big O for the models applied here is very challenging, we try to provide a rough estimate and qualitative comparison.

In the case of ELM, the computational complexity is relatively lower than other applied techniques. It usually depends on the number of neurons (N_n_) and data dimension (D_d_). In this regard, the computational complexity of approximately O (N_n_ × D_d_) may be assumed. The computational complexity of ANFIS is influenced by a combination of factors, including the number of fuzzy rules, the selected training method, the complexity of membership functions, and the quantity of input variables. In general, ANFIS models tend to have a moderate level of computational complexity.

Nevertheless, it is essential to recognize that the exact complexity can vary considerably based on the specific configuration and the nature of the problem under consideration^72^. Therefore, assigning a big O is very challenging. Like ANFIS, The computational complexity of MLP can range from moderate to high, depending on their specific configurations.

The computational complexity could be approximately on the order of O (K_e_ × N_n_ × D_d_), with K representing the count of training epochs. The computational complexity of an RBF network is influenced by factors like the number of RBF units, the dimensionality of the input data, and the training procedure. In the training phase, an overall computational complexity of approximately O (M × N × D_d_ × I) is assumed, where M refers to the training sample size, N represents the number of RBF units, and I indicates the number of iterations necessary for reaching convergence. However, some references state the RBF complexity simply as O(N^2^)^73^.

### Model trend estimation

**Figure 9:**
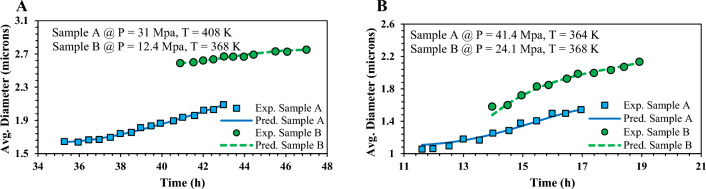
Comparison of predicted and experimental asphaltene aggregate diameter versus time for oil samples A and B at different temperature and pressure values.

Trend estimation is a crucial step in post-evaluating a modeling strategy and verifying a model's ability to track the variations in experimental measurements. To validate the robustness of the best-proposed model, we assessed the GWO-RBF approach's trend-prediction capability under different conditions, as shown in Fig. 9. The figure presents a comparison between the predicted and experimental average diameter of asphaltene aggregates over time for oil samples A and B under varying pressure and temperature conditions. The figure demonstrates that the GWO-RBF model accurately predicts the asphaltene aggregate diameter and the process trend under different states.

This study offers an AI-based predictive framework for accurately predicting the kinetics of asphaltene particle aggregation, addressing a critical concern in different phases of production from petroleum reservoirs. Its effectiveness lies in the accuracy of predictions obtained through the utilization of diverse AI methodologies and the thorough examination of significant variables. In addition, the proposed strategies have the potential to be integrated with the available simulator to consider the asphaltene precipitation phenomena more accurately. This integration could result in mitigating the uncertainties and improving prediction capability. Nonetheless, the AI-based paradigms are usually restricted by their reliance on available data and potential challenges in generalizing to a wide range of conditions. In other words, the extent of practical applicability of the developed models could be improved as more data is available for their training.

## Conclusions

In the current research, reliable models based on GWO-RBF, MLP-LM, MLP-BR, MLP-SCG ANFIS, and ELM were constructed to estimate the asphaltene aggregate diameter versus time in terms of pressure, temperature, oil asphaltene content, and oil specific gravity. A series of experimental data was acquired based on the published literature to construct the model. A terrific match was obtained in terms of statistical parameters between the experimental and predicted values of asphaltene aggregate diameter using the developed models. However, the proposed GWO-RBF model exhibits higher accuracy compared to the other models for which the resulted coefficient of determination, average absolute relative deviation percent, and root mean square error were 0.9993, 1.1326%, and 0.0537, respectively. The obtained results show the strong generalization ability and high prediction capability of the introduced models.

## Data Availability

The datasets generated during and/or analyzed during the current study are available from the corresponding author on reasonable request.
